# Letter from Dr. W. H. Dwinelle

**Published:** 1847-06

**Authors:** W. H. Dwinelle


### ARTICLE IV.

### *Letter from* Dr. W. H. Dwinelle.

Messrs. Editors:

There seems to be some misunderstanding in reference to an article published under my name, in the September number, 1846, of the Journal.

The article was somewhat hastily written, or I should not have fallen into the error of using terms so inexplicit, and capable of such various construction, without the safeguard of further qualification.

The term decay is susceptible of so many significations, that, even in ordinary usage, it is exceedingly ambiguous; out of this circumstance the misapprehension seems to have arisen.

I did not intend to be understood as advocating the propriety of leaving *actual* decay or *decomposed* matter in the cavity of the tooth; I mean in the strict sense of the term. But I *did* mean to be understood to say, that there are circumstances wherein it is proper, and even *duty*—rather than do worse—to have discolored matter in the cavity of the tooth, and even those parts of the tooth which have lost a portion of its lime, and with it a degree of its density. Because, in the first place, a tooth may be discolored, and yet retain all its original qualities of density. And, in the second place, a tooth may lose a fraction of its lime, and yet retain all its essential qualities as a bone, as is manifest in the bones of different individuals, some of which almost vie with flint in texture, while others again may be literally whittled with a knife. The bones of an infant contain but comparatively a small quantity of lime, and yet they possess all the essential qualities of bone—distinct, living, organized bone. The bones of an aged person contain a much less quantity of gelatine, and yet they perform all the requisite functions of bone.

That I may not again be misunderstood, let me introduce an illustration: The bones are composed of lime and animal matter, suppose we say five parts of animal matter to ten of lime; suppose the decomposing influence of acids have deprived it of one or two proportions of its lime, still it is bone in all its essential qualities, and, if properly protected from external influences, will answer all the requisite properties of bone; the decomposition, under the circumstances, will not go on, nor will it dry down, and occupy less space than it did before, as *cartilage* would, situated in a dry place, and entirely remote from the influence of moisture. It would not dry down, I say, especially as the tooth is not dead, and as the whole surrounding bone is of a porous quality, and the parts are immediately over a moist, living nerve; the parts have lost a small portion of its lime, and have assumed a modified character of original bone; enough has been lost to render the change discernible, and yet they are sufficiently dense and firm to resist the force of a proper plugging.

The word *decompose*, as well as decay, admits of several significations, and can be *tortured* into many; but I wish to be understood as using it in view of the circumstance, in the light of common sense, and in a comparative and qualified degree, starting at a point at which its action is imperceptible, it passes down, step by step, one degree after another, until its operation is complete. The very term denotes *progressive action*, as it is, indeed, a chemical action; so that the idea of disintegration is wholly incompatible with the premises.

My object in writing the article, was to endeavor, so far as possible, to correct that which I am compelled to believe a morbid disposition on the part of some—irrespective of all qualifying circumstances—to pursue and exterminate all discolored and slightly modified bone, even to the nerve, and then tell us how skilfully and ingeniously they have killed and removed a living and valuable organization, as though it was a guilty thing—much in the same way that one headsman would boast to another, how handsomely and artistically he had decapitated a fellow being. For I lay it down as a fact beyond controversy, that the nerve of a tooth is essential to its perfect and healthful existence, and firmly believe that when it is protected by discolored and slightly decomposed bone, and which bone cannot be removed without exposing it, it is infinitely better to proceed and fill the tooth, rather than remove the nerve, thereby robbing the tooth of a large share of its necessary vitality. The destruction of the nerve is oftentimes important and imperative, but very, very often it is not.

I trust I am more fortunate in being understood this time, and also hope, as I had hoped before, that I may thereby be instrumental in promoting the general good of the profession, yet I may be mistaken; "Tempus discernet."

Ever truly, your friend,

W. H. DWINELLE.

